# Jackals as carriers of *Leishmania *and *Brucella *species in Serbia

**DOI:** 10.1186/1756-3305-7-S1-P19

**Published:** 2014-04-01

**Authors:** D Ćirović, D Chochlakis, S Tomanović, R Sukara, A Penezić, Y Tselentis, A Psaroulaki

**Affiliations:** 1 Department for Animal Ecology and Geography, Faculty of Biology, University of Belgrade, Serbia; 2 Regional Laboratory of Public Health of Crete, Heraklion, Crete, Greece; 3 Laboratory for medical Entomology, Institute for medical Research, University of Belgrade, Serbia; 4 Laboratory of Clinical Bacteriology, Parasitology, Zoonoses and Geographical Medicine, University of Crete, Heraklion, Crete, Greece

## 

The golden jackal *Canis aureus *occurs in south-eastern Europe, Asia, the Middle East, the Caucasus and Africa as well as in countries of the European continent, including Serbia. The scope of the current survey was to study the presence of two zoonotic agents, *Leishmania *species (vector transmitted) and *Brucella *species, in the jackal population in Serbia.

Animal samples were collected over a three-year period (01/2010 - 02/2013) from 48 localities corresponding to 12 regions of Serbia. Sampling took place from dead animals brought at the laboratory of Department for Animal Ecology and Geography, Faculty of Biology, University of Belgrade, by hunters. Data regarding location were recorded. At necropsy, samples from different tissues were collected (spleen, liver, hart); spleen was chosen as the tissue to work with. Samples were homogenised with micropastles (Eppendorf). DNA extraction took place from a small portion of smashed tissue using the Gene Jet Genomic DNA Purification Kit, (Fermentas, Thermo Scientific). For the detection of *Brucella *species, a multiplex Real-time PCR protocol was used, targeting the *bcsp31*, *alkB *and *BMEI1162 *genes of *Brucella *species, *B. abortus *and *B. melitensis*; *B. canis *was detected using a PCR protocol targeting the *omp2B *gene. For the detection of *Leishmania *species a Real-time PCR protocol targeting the SSU rRNA gene was used.

A total of 216 spleen samples were tested. Most samples (196/90.7%) were collected during the hunting period (November-February). Of the samples tested, 108 animals were male and 108 were female. Fifteen (6.9%) were positive for *Leishmania *species, while four (1.9%) were positive for *B*. *canis*.

Jackals are known omnivores and scavengers and they usually prey on small mammals, on vegetables, fruit and garbage. Their ability to adapt to novel habitats both on rural and urban areas allows them to come into contact with animals that live in close proximity to humans, such as dogs, hence their ability to host a number of zoonotic, including vector-borne, pathogens. An increase in jackal population has lately been observed in Serbia, while complaints about damages that jackals may cause to livestock mean that their wildlife cycle may well interfere with areas of natural human activity. Although further studies are required to discern the potential epidemiologic role of the golden jackal in spreading and transmitting the above studied pathogens, local awareness of residents, veterinarians, and health professionals would prove of great importance in order to efficiently monitor these and other zoonotic diseases.

